# Modified distance regularized level set evolution for brain ventricles segmentation

**DOI:** 10.1186/s42492-020-00064-8

**Published:** 2020-12-07

**Authors:** Thirumagal Jayaraman, Sravan Reddy M., Manjunatha Mahadevappa, Anup Sadhu, Pranab Kumar Dutta

**Affiliations:** 1 School of Medical Science and Technology, IIT Kharagpur, Kharagpur, 721302 India; 2 Department of Electronics and Communications, JNTUA-College of Engineering, Pulivendula, 516390 India; 3 EKO CT & MRI Scan Centre, Medical College, Calcutta, 700073 India; 4 Department of Electrical Engineering, IIT Kharagpur, Kharagpur, 721302 India

**Keywords:** Ventricles, Atrophy, Segmentation, Level set, Diagnosis

## Abstract

Neurodegenerative disorders are commonly characterized by atrophy of the brain which is caused by neuronal loss. Ventricles are one of the prominent structures in the brain; their shape changes, due to their content, the cerebrospinal fluid. Analyzing the morphological changes of ventricles, aids in the diagnosis of atrophy, for which the region of interest needs to be separated from the background. This study presents a modified distance regularized level set evolution segmentation method, incorporating regional intensity information. The proposed method is implemented for segmenting ventricles from brain images for normal and atrophy subjects of magnetic resonance imaging and computed tomography images. Results of the proposed method were compared with ground truth images and produced sensitivity in the range of 65%–90%, specificity in the range of 98%–99%, and accuracy in the range of 95%–98%. Peak signal to noise ratio and structural similarity index were also used as performance measures for determining segmentation accuracy: 95% and 0.95, respectively. The parameters of level set formulation vary for different datasets. An optimization procedure was followed to fine tune parameters. The proposed method was found to be efficient and robust against noisy images. The proposed method is adaptive and multimodal.

## Introduction

The brain gets affected by multiple factors; degenerative disorders are one of them. Degenerative disorders could be a result of environment changes, food habits, work depression, hereditary, traumatic injury or caused from the effect of other neurological disorders. Degenerative disorders in the brain render the subject unable to perform their activities of daily living such as communication, movement, balancing their body while walking and sometimes vital functions such as respiration and functioning of the heart. Atrophy in the brain or cerebral atrophy is a symptom or a form or stage which is caused by many diseases. In atrophy, the brain starts to shrink because of neuronal loss [1, 2]. Atrophy affects all structures of the brain, but in different ratios. Therefore, for the diagnosis of atrophy, certain regions or structures of the brain are preferred by experts. Atrophy can be detected by several ways: mini mental state exam, electroencephalography, using imaging modalities such as computed tomography (CT), magnetic resonance imaging (MRI), positron emission tomography (PET) and single photon emission computed tomography (SPECT), measuring cerebrospinal fluid (CSF) pressure and biopsy of the brain tissues. Imaging modalities give an advantage of a clear view of the brain and helps in giving the location and structure which has more atrophy in terms of quantification [3, 4]. This will aid the physician in early detection.

The ventricular system is one of the prominent structures in the brain, located in the mid-regions of the brain, filled with CSF [5]. At different stages of degenerative disorders, atrophy in particular, the volume of the CSF tends to increase due the shrinkage or loss of neurons [5, 6]. As ventricles are filled with CSF, their shape and size also keep changing due to the changes occurring in the CSF and the subsequent progression of the disease [1, 4]. Thus, brain ventricles serve as a major landmark or biomarker for detecting atrophy. Anandh et al. [7] attempted a ventricular enlargement study to differentiate between mild cognitive impairment (MCI) and Alzheimer’s disease. This group employed shape descriptors to study the shape differences of ventricles and used it for the classification purpose. Wang et al. [8] used the shape of the brain ventricles for improving the classification accuracy of Alzheimer’s disease from normal subjects and other diseases using shape descriptors. Bader et al. [9] performed volume estimation of CSF and ventricles, to verify correlations between CSF oscillations and ventricular dilatation, and how their variations are indicated in brain disorders. An experimental based structural mapping was employed by Carmichael et al. [10] for distinguishing dementia and MCI in a large community.

For landmark based diagnostic systems such as morphometric and volumetric studies, the region of interest needs to be either highlighted or separated from the background [11–13]. Hence object segmentation is a pre-processing step in a diagnostic system. The manual segmentation process is labor intensive, time consuming, and could be biased, and its accuracy is based on the individual’s training skills. Thus, computer-based segmentation procedures are preferred by experts to minimize manual errors. It is important that these computerized algorithms are accurate, rapid, cost effective, automatic, and robust [13].

There are different types of segmentation techniques that have been reported in the literature: thresholding-based techniques, region growing and splitting algorithms, clustering techniques, atlas-based models, deformable models, and neural networks and classifiers [14]. Baghdadi et al. [15] came up with the Bayesian Generalized Fast Marching algorithm for segmenting brain structures including the ventricles. Mishra et al. [14] presented a hybrid segmentation procedure using the watershed algorithm and distance regularized level set evolution (DRLSE) algorithm for segmenting gray matter, white matter and CSF from MR images of the brain. Xia et al. [12] developed a knowledge-driven algorithm for automatic segmentation of the ventricles from MR images. Narr et al. [16] reported a 3D mapping of temporo-limbic regions and ventricles for schizophrenic subjects. Anandh et al. [17] incorporated Tukey’s bi-weight edge indicator into a level set formulation to segment the ventricles in Alzheimer’s subjects and the method was shown to be clinically significant. Angelini et al. [18] implemented a four phase, 3D active contour model on 3D images for partitioning brain MR images. This group demonstrated that, the proposed framework was efficient in segmenting the regions of interest and flexible with the set of features. Aloui and Naceur [19] proposed an approach based on level set method for delineating tumor, followed by mesh simplification to separate them out.

Even though there are many techniques available, the technique chosen varies between organs, region of interest, depending on the disease, subject’s age, imaging modality (X-ray, CT, MRI, PET, SPECT), method of visualization, features to be used from the segmented image [1, 2]. Hence adaptive and multi-modal segmentation algorithms need to be developed [19]. This study employs a modified DRLSE with optimized parameters for segmenting ventricles from brain images [20, 21]. The objective of this study was to segment the ventricles based on a modified level set technique to improve segmentation accuracy and diagnosis involving ventricles.

## Methods

The methods section is divided into two parts: optimization of parameters and main segmentation framework.

### Level set formulation

The level set method was first introduced by Stanley Osher and James Sethian in the 1980s [22]. It is based on the active contour model concept, where the curves are represented as zero level set, with a function of higher dimension, which is called as the level set function (LSF) [22, 23]. The curve evolves, depending on certain energy functions from the image, which are derived by partial differential equations. The energy functions give the speed at which the curve evolves and result in a smoothing process. The stopping point of the curve evolution is the boundary of the object to be segmented [3, 24]. The extraction of the curve is dependent on the initial parameters chosen by the user. For this reason, LSF’s performance relies more on the initialization of the parameters. There have been different re-initialization procedures, regularization procedures reported in literature for making LSF efficient and accurate [25, 26]. It can represent shapes having complex topology [27, 28]. The LSF is defined as follows: parametric contour is given by ${C}_1\left(s,t\right):\left[0,t\right]\times \left[0,\infty \right]\to {\mathfrak{R}}^2$, which defines the parameterization of a point in the contour [22, 23, 26]. The curve evolution is defined by the following equation

1 $$ \frac{\partial {C}_1\left(s,t\right)}{\partial t}=\mathcal{F}N $$

*C*_1_ is curve, $\mathcal{F}$ is speed function that controls the motion of the contour, *N* is inward normal vector to the contour. The level set surface needs to remain with the same smoothness level; hence initialization has to be given at regular intervals - this process is called as re-initialization [29, 30].

2 $$ {\phi}_t=\mathit{\operatorname{sign}}\left({\phi}_0\right)\left(1-|\nabla {\phi}_0|\right) $$

∇ is the gradient operator of the image and *ϕ* is the LSF that needs to be re-initialized.

### Optimization of parameters

Even though the level set method has many advantages, it has a drawback in the form of re-initialization; which leads to increased complexity in terms of user intervention and execution time is more. Therefore, Li et al. [21, 22] proposed DRLSE method which eliminated the need for re-initialization process. The set of input parameters used in their study were not able to segment the brain ventricles correctly for the dataset used in this study. Therefore, a preliminary study of optimizing the parameters was implemented for input images. An optimization procedure was followed for all the images (*n* = 80 subjects): 20 atrophy CT subjects, 20 atrophy MRI subjects, 20 normal CT subjects, and 20 normal MRI subjects. In selective optimization images were included randomly from the study groups (2 atrophy and 3 normal subjects from CT dataset, 3 atrophy and 2 normal subjects from MRI dataset). The parameters which were tuned were alpha (α), lambda (λ), epsilon (ε), time step (∆t), edge kernel (Gaussian, Average, Median), kernel size and sigma value; whose significance will be explained later. As given in the algorithm the value which produces highest accuracy value when compared with ground truth image is considered to be the tuned value for the respective parameter. This is explained in the results section with a sample table (Table 1). The tuned parameters are common for both the datasets used in this study; CT and MR images as the optimization procedure was carried out commonly for both the datasets (mingling images from CT and MR, making it into a single group and then carrying out optimization procedure).

**Table 1:** Average and SD values of similarity measures between ground truth and segmented results (*n* = 40)

Epsilon	DI	JI	Sensitivity	Specificity	Accuracy
0.5	0.55 ± 0.0148	0.38 ± 0.0112	87.8 ± 0.61	96.4 ± 0.72	96.2 ± 0.62
1	0.53 ± 0.0217	0.41 ± 0.0254	88.6 ± 0.45	96.5 ± 0.5	96.3 ± 0.62
1.5	0.52 ± 0.0356	0.39 ± 0.034	90.4 ± 0.78	96.5 ± 0.52	96.3 ± 0.72
2	0.59 ± 0.0294	0.46 ± 0.029	90.6 ± 0.86	96.59 ± 0.73	96.39 ± 0.54
2.5	0.56 ± 0.0378	0.39 ± 0.034	90 ± 0.6	96.5 ± 0.63	96.3 ± 0.42

**Fig. 1:**
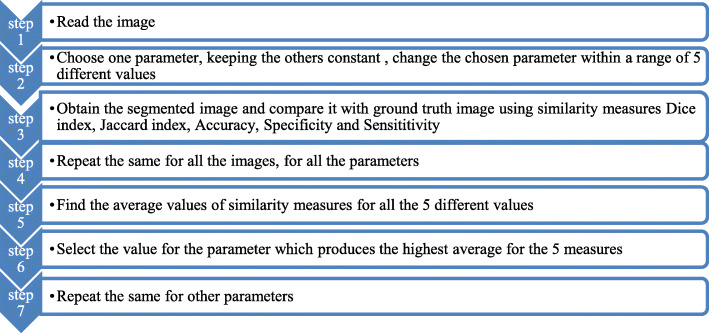
The flowchart for parameter optimization

There are many optimization techniques available: linear, nonlinear, grid search, Bayesian, random search, and gradient based to make this parameter tuning generalized. Generalization means, parameter optimization for the segmentation framework such that, it performs well on all the datasets - irrespective of the geographical region, age and gender of the subject, imaging properties and imaging modality (The optimization carried out in this paper is specific for the datasets used for this study). However, this involves the appropriate selection of the optimization technique based on the data and dimension of the parameters, training and testing of the parameters, cross validation, etc. This will make the selection of the parameter computationally complex. The objective of this study was to improve the DRLSE method; the proposed modification was made to make it suitable for segmenting the input images. Moreover, the parameters used in the original DRLSE study for their synthetic data were found to be unsuitable for the input data used in this study, thereby leading to failure of the segmentation procedure or improper segmentation. Therefore, this study used a simple optimization procedure for selecting suitable values for the parameters as shown in Fig. 1. If we need to go for a generalization of the parameter selection that will make the entire algorithm complex, as the segmentation algorithm itself takes up to 2 min for execution. The algorithm is as follows (Fig. 1). The tuned parameter values are: alpha-2, lambda-1, epsilon-2, time step-1.5, edge kernel-Gaussian, sigma for Gaussian kernel-2.5, size of the kernel-15.

### DRLSE

Li et al. [20, 21] proposed the DRLSE method to overcome the trouble of re-initialization. In DRLSE the surface regularity of the LSF is intrinsically maintained by a regularizing term. Hence with the surface fitting energy term, there will be a regularization term in the LSF [31]. The initial LSF, gradient operator and signed distance function are the same as the conventional level set method and have been adapted in DRLSE. Due to space constraint only; the relevant equations are given here. The remaining equations and their derivations can be referred from refs. [20, 21, 31]. The energy term is given as follows

3 $$ {\varepsilon}_{\varepsilon}\left(\varnothing \right)=\mu {\int}_{\Omega}p\mid \nabla \varnothing \mid dx+\lambda {\int}_{\Omega}g{\delta}_{\varepsilon}\left(\varnothing \right)\mid \nabla \varnothing \mid dx+\alpha {\int}_{\Omega}g{H}_{\varepsilon}\left(-\varnothing \right) dx $$

Where, *ε*_*ε*_ (∅) is energy functional, *ε* is a constant, the first term is level set regularization term and *μ* > 0 is a constant, *p* is potential function, *λ* > 0 coefficient of the area term: the second term in the equation, *α* is coefficient of weighted area term, it should be positive so that zero level contours can shrink in the level set evolution. The role of *g* in the energy term (area term) is to slow down the shrinking and expanding of the zero level contours when it arrives at object boundaries where *g* takes smaller values. The last term is the line integral, *δ* and *H* are Dirac delta function and Heaviside functions respectively [20, 24, 32]. Applications to image segmentation using edge information are obtained from the image

4 $$ g\triangleq \frac{1}{1+\mid \nabla {G}_{\sigma}\ast I\mid \hat{\mkern6mu} 2} $$

*G* stands for Gaussian kernel, *I* is the image and *σ* is the standard deviation (SD) of the kernel, which defines the blurring of Gaussian kernel. The convolution is used for smoothening the image to reduce noise.

### Region-scalable fitting model

The region-scalable fitting (RSF) model uses the region based active contour model to provide information on the low-intensity pixels. A fitting energy is obtained from the region of interest in the image based on pixel intensities [33]. This fitting energy is divided into two functions: one defines the intensity information inside the boundary and the other approximates the region outside the boundary [34–36]. Due to space constraint only, the relevant equations are given here as it will be a repetition of what is already present in ref. [35]. The remaining equations and their derivations can be referred from ref. [35]. For an image *I*(*x*, *y*), the energy functional is

5 $$ {\mathcal{F}}^{MS}\left(u,C\right)={\int}_{\Omega}{\left(u-I\right)}^2 dx+\mu {\int}_{\Omega /c}{\left|\nabla u\right|}^2 dx+v\left|C\right| $$

|*C*| is the length of contour *C*, *I* is the input image, *u* approximates the image *I*, *μ* is a constant, Ω is the image domain and *ν* is a constant. The energy function is derived by minimizing Mumford –Shah function for a curve. It can be rewritten as follows for a given point *x* ∈ Ω

6 $$ {\varepsilon^{fit}}_x\left[C,{f}_1(x),{f}_2(x)\right]=\sum \limits_{i=1}^2{\lambda}_i{\int}_{\Omega}{K}_{\sigma}\left(x-y\right){\left|I(y)-{f}_i(x)\right|}^2\ast {M}_i\left[\varnothing (y)\right] dy $$

*ε*^*fit*^_*x*_= fitting energy, *λ*_1_ and *λ*_2_ are positive constants, *f*_1_(*x*) and *f*_2_(*x*) are two values that approximate image intensities in domains Ω_1_ and Ω_2_. *I*(*y*) = image intensities involved in the above fitting energy. *K*_*σ*_ is non negative kernel function.

### Modified DRLSE

**Fig. 2:**
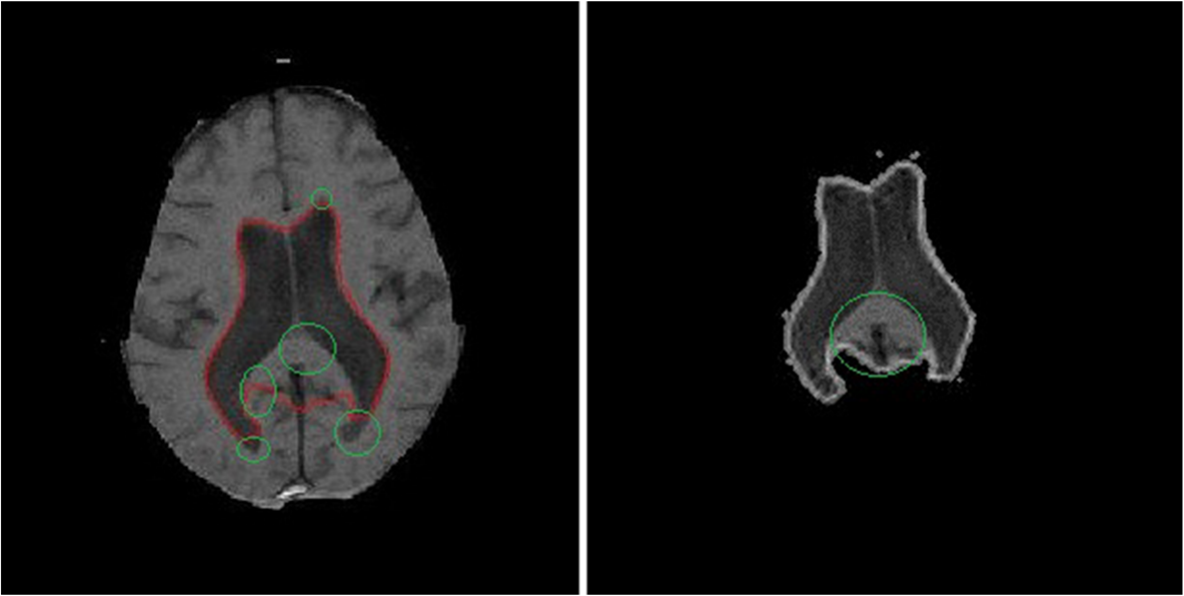
Resultant images of segmentation using the conventional DRLSE method. The incorrect segmented regions are marked in green color circles

**Fig. 3:**
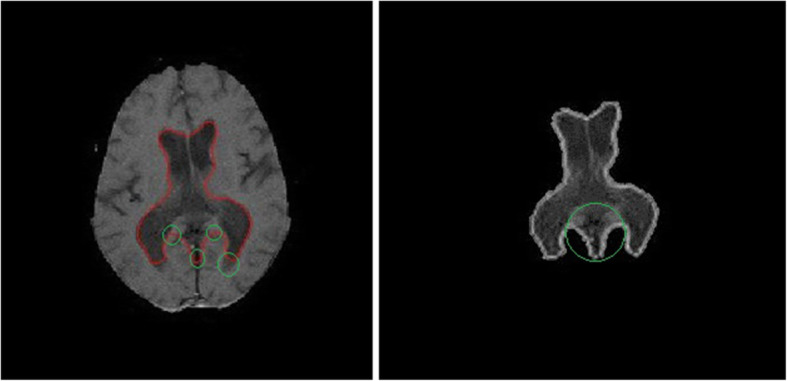
Resultant images of segmentation using the conventional DRLSE method with optimized parameters. Without the regional information, the DRLSE framework fails to converge to the actual boundary

DRLSE has many advantages compared to the conventional level set method, in terms of initialization. However, it lacks in one feature that it uses only the edge information from the input image. This leads to incorrect segmentation of the ventricles, as it has both sharp and weak boundaries present at different regions of the ventricles as shown in Fig. 2. Even with the optimized parameter, it fails to converge sometime as shown in Fig. 3. Wherever a weak boundary is present with small change in the intensities, it was not well detected by the edge indicator as it considers only gradient changes, eventually leading to incorrect segmentation. DRLSE needs to have a term that accounts for pixel wise intensity information from the images which will detect very small changes in the intensity such as weak boundaries. Therefore, this study proposes the incorporation of the RSF concept into DRLSE for better segmentation.

RSF-based segmentation works well in case of weak boundaries as it accounts for regional (pixel wise information in a particular region) intensity information but suffers from re-initialization [34–36]. RSF does not account for any edge information; sometimes it fails to converge and suffers from re-initialization. A study has been reported in the literature where DRLSE was applied first on the image, followed by RSF; which merely increases the computational complexity and time [37].

Therefore, this study proposes to incorporate regional intensity approximations of two regions from the RSF to the DRLSE framework which already has edge indicator and the regularization term. This way, the LSF will have region based and boundary based fitting energy term and a regularization term to avoid the complex re-initialization process. This reduces the computational complexity as there is only intensity information incorporated into the DRLSE equation instead of merely adding it to the framework, less computational time and efficient segmentation. This proposal is made to overcome the limitations of the DRLSE method. The proposed framework is given as follows (only the final equations combining both the frameworks are presented here as other equations and terms have been presented in the previous sections): Energy Functional equation is obtained by combining DRLSE and RSF. The RSF term is

7 $$ \varepsilon \left(\varnothing \right)={\lambda}_1{\int}_{\Omega}{K}_{\sigma}\left(x-y\right){\left|I(y)-{f}_1(x)\right|}^2\ast {M}_1\left[\varnothing (y)\right] dy+{\lambda}_2{\int}_{\Omega}{K}_{\sigma}\left(x-y\right){\left|I(y)-{f}_2(x)\right|}^2\ast {M}_2\left[\varnothing (y)\right] dy $$

The DRLSE equation for LSF

8 $$ {\varepsilon}_{\varepsilon}\left(\varnothing \right)=\mu {\int}_{\Omega}p\mid \nabla \varnothing \mid dx+\lambda {\int}_{\Omega}g{\delta}_{\varepsilon}\left(\varnothing \right)\mid \nabla \varnothing \mid dx+\alpha {\int}_{\Omega}g{H}_{\varepsilon}\left(-\varnothing \right) dx $$

Approximations for line and area term in the DRLSE framework

9 $$ {\lambda}_1{\int}_{\Omega}{K}_{\sigma}\left(x-y\right){\left|I(y)-{f}_1(x)\right|}^2\ast {M}_1\left[\varnothing (y)\right] dy\cong \lambda {\int}_{\Omega}g{\delta}_{\varepsilon}\left(\varnothing \right)\mid \nabla \varnothing \mid dx $$

10 $$ {\lambda}_2{\int}_{\Omega}{K}_{\sigma}\left(x-y\right){\left|I(y)-{f}_2(x)\right|}^2\ast {M}_2\left[\varnothing (y)\right] dy\cong \alpha {\int}_{\Omega}g{H}_{\varepsilon}\left(-\varnothing \right) dx $$

The final equation by combining (8), (9) and (10).

11 $$ \varepsilon \left(\varnothing \right)=\mu {R}_p\left(\varnothing \right)+\lambda {\int}_{\Omega}g{\left|I(y)-{f}_1(x)\right|}^2\ast {M}_1\left[\varnothing (y)\right] dy+\alpha {\int}_{\Omega}g{\left|I(y)-{f}_2(x)\right|}^2\ast {M}_2\left[\varnothing (y)\right] dy $$

**Fig. 4:**
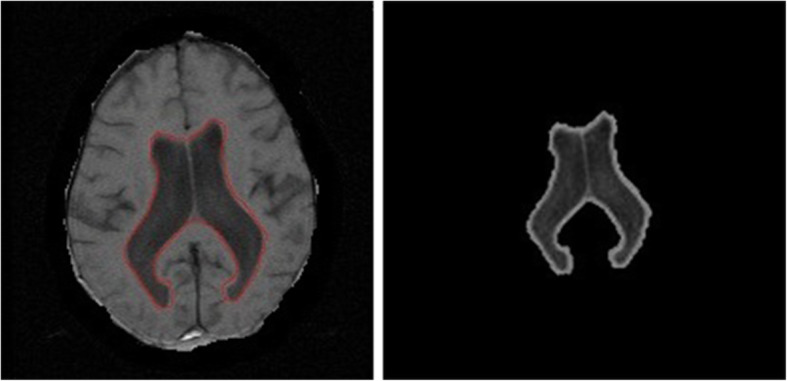
Resultant images of segmentation using the proposed method with optimized parameters. With the regional information, the proposed method evolves to the actual boundary of the object

**Fig. 5:**
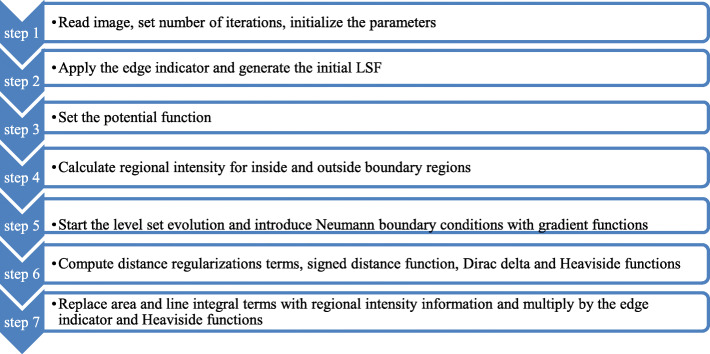
Flow chart explaining the numerical implementation of the proposed method

Where M_1_ and M_2_ are the equivalents of Heaviside and Dirac delta functions presented in the second and third terms of Eq. (8), which were applied in the final computation. In Eq. (11), the edge indicator term g is from the DRLSE framework, *g*|*I*(*y*) − *f*_1_(*x*)|^2^ ∗ *M*_1_[∅(*y*)]*dy* is the regional intensity approximation from the RSF framework from domain 1 (inside the boundary), and *g*|*I*(*y*) − *f*_2_(*x*)|^2^ ∗ *M*_2_[∅(*y*)]*dy* is the regional intensity approximation from the RSF framework from domain 2 (outside the boundary) along with the edge indicator. The edge indicator of the DRLSE model is used here (*g* = edge indicator). Figure 4 shows the segmented ventricles using the proposed method. Step by step implementation of the proposed algorithm is shown in flowchart in Fig. 5.

12 $$ {K}_{\sigma}\left(x-y\right)=g $$

## Results

Twenty CT images and twenty MRI T1 weighted images of healthy subjects were considered. Twenty CT images and twenty MRI T1 weighted images of atrophy subjects were included in the dataset. The CT images chosen were of axial slices with slice thickness 5 mm and size of 512 × 512 pixels. The MRI images used were of axial slices with slice thickness 5 mm and size of 256 × 256 pixels. The images were collected from Calcutta Medical College, Kolkata, West Bengal, in digital imaging and communications in medicine format. The atrophy subjects were chosen by the expert radiologist and were confirmed by a clinical diagnostic process. Each brain volume was skull stripped using an algorithm which involves binarization, labeling, finding region properties and morphological operations. The segmented results were validated with ground truth images, made with the help of an expert radiologist. The brain images of respective slices were edited using image editing software; the borders of the ventricles were traced manually. After drawing the borders manually, the images were shown to the radiologist to verify the accuracy. Then, the regions inside the borders were extracted using MATLAB coding. These were used as ground truth images for comparison with the segmented images. Then the modified DRLSE method was applied as shown in Fig. 5.

### Results of optimization of parameters

Table 1 shows the results of the optimized parameter for the input images. The segmented results were compared with the ground truth images using sensitivity, specificity, accuracy, Dice index, and Jaccard index. The average values of sensitivity, specificity, accuracy, Dice index, and Jaccard index for the input images were obtained and that producing higher values was chosen to be the optimized value of that parameter for segmentation. The Jaccard similarity index between two images A and B was defined as

13 $$ \mathrm{JI}=\frac{\left|\mathrm{A}\cap \mathrm{B}\right|}{\left|\mathrm{A}\cup \mathrm{B}\right|} $$

The Dice similarity coefficient between two images A and B was defined as

14 $$ \mathrm{DI}=2\ast \frac{\left|\mathrm{A}\cap \mathrm{B}\right|}{\left|\mathrm{A}\right|+\left|\mathrm{B}\right|} $$

In case of the parameter epsilon which is used in the Heaviside function, epsilon-2 produced better results than other values for all the five measures (0.5 to 2.5) as highlighted in the table.

### Segmentation results

#### Statistical analysis

Sensitivity, specificity and accuracy, peak signal to noise ratio (PSNR) and structural similarity index measure (SSIM) were used as performance measures. The formulae for these measures are as follows

15 $$ \mathrm{sensitivity}=\frac{TP}{TP+ FP} $$

16 $$ \mathrm{specificity}=\frac{TP}{TN+ FP} $$

17 $$ \mathrm{accuracy}=\frac{TP+ TN}{T\mathrm{P}+ FN+ TN+ FP} $$

18 $$ \mathrm{PSNR}=10\ {\log}_{10}\left(\frac{peakvaluve^2}{meansquarederror}\right) $$

**Fig. 6:**
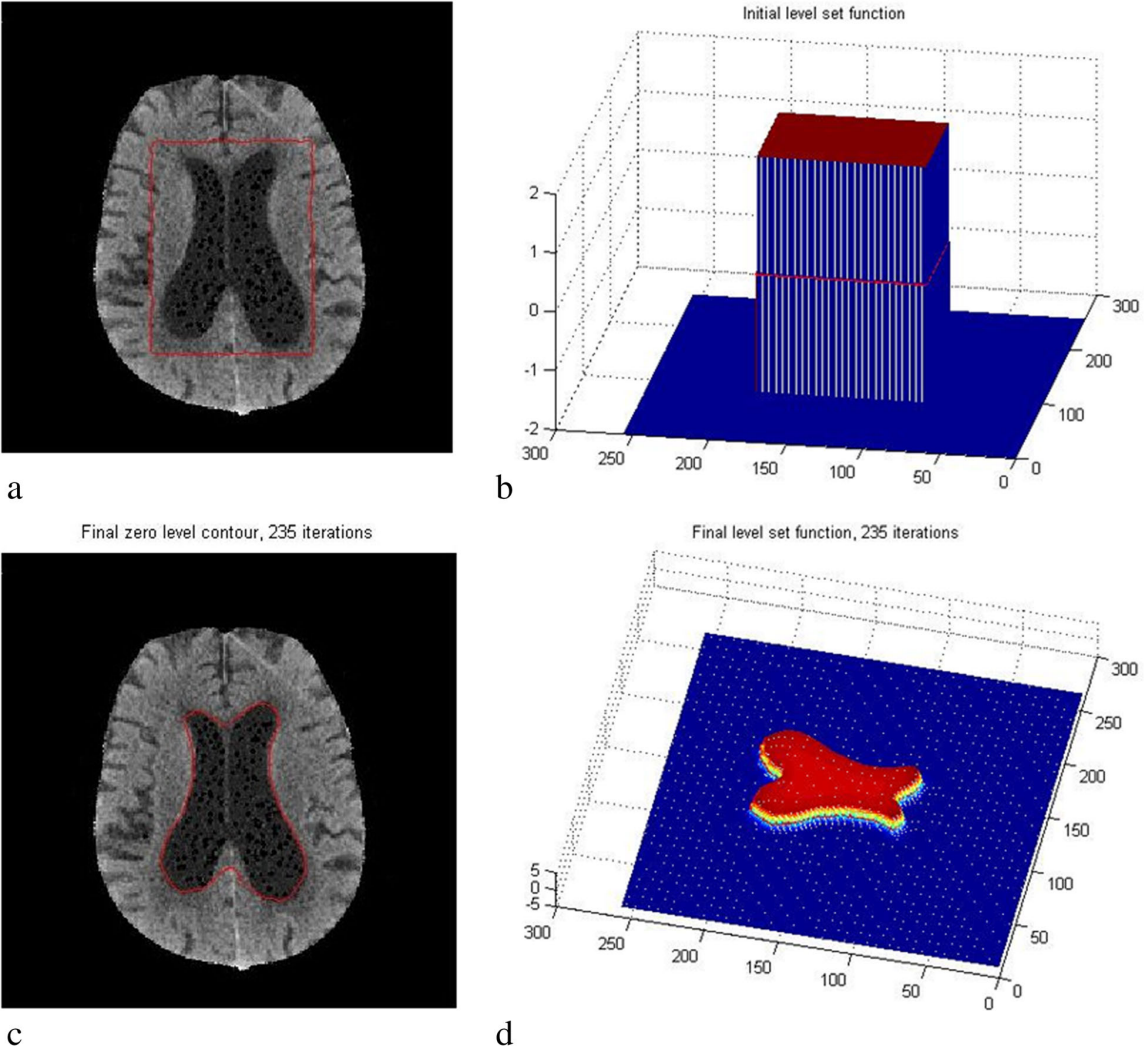
Resultant images of the proposed level set segmentation showing **a** initial bounding box, **b** initial LSF, **c** segmented ventricles; boundary marked in red color after iterations and **d** LSF of the segmented ventricles

True negative (TN) is pixels correctly detected as background, true positive (TP) is pixels correctly segmented as foreground, false negative (FN) is pixels falsely detected as background, and false positive (FP) is pixels falsely segmented as foreground. Figure 6 shows the resultant images of segmentation using the proposed method. Figure 6a shows the initial bounding box around the ventricles in the input image. Figure 6b shows the LSF for the initial bounding box. Figure 6c shows the final zero level contour of the object of interest after 236 iterations. Figure 6d shows the final LSF for the segmented object from the image after 236 iterations. This figure was included in the paper to show the steps involved in the segmentation process in terms of figures for the image and LSF.

**Fig. 7:**
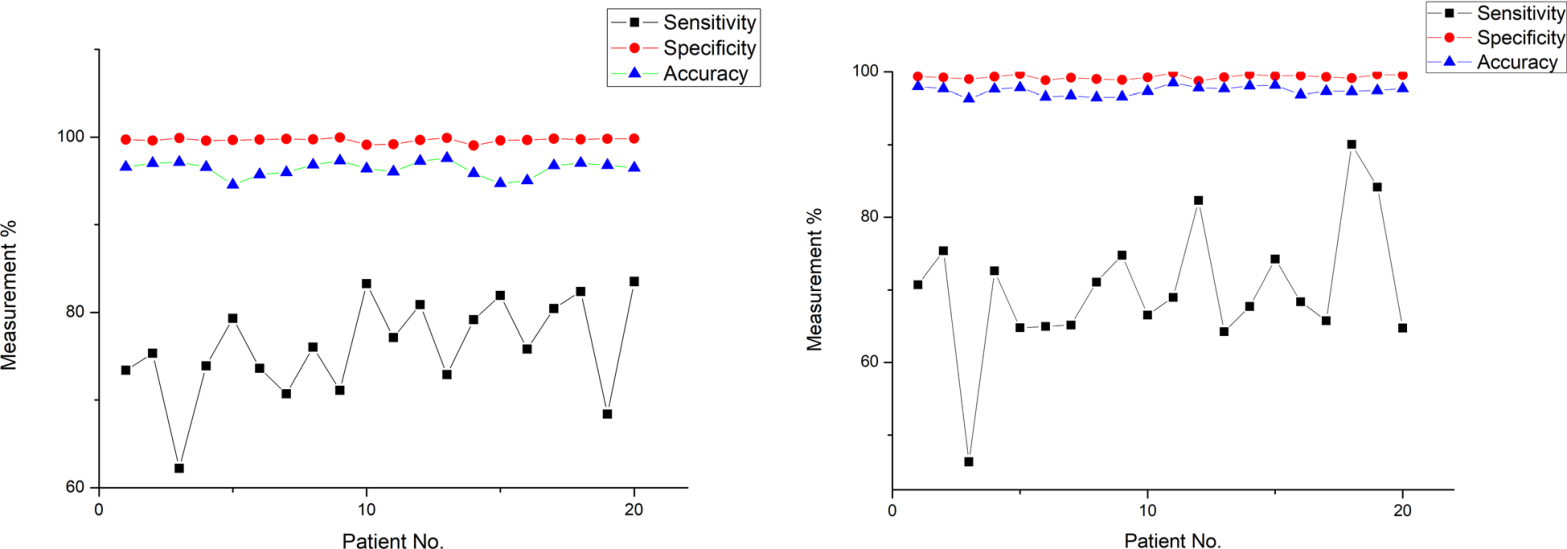
Results of sensitivity, specificity and accuracy for CT dataset using the modified DRLSE for atrophy and normal subjects

**Fig. 8:**
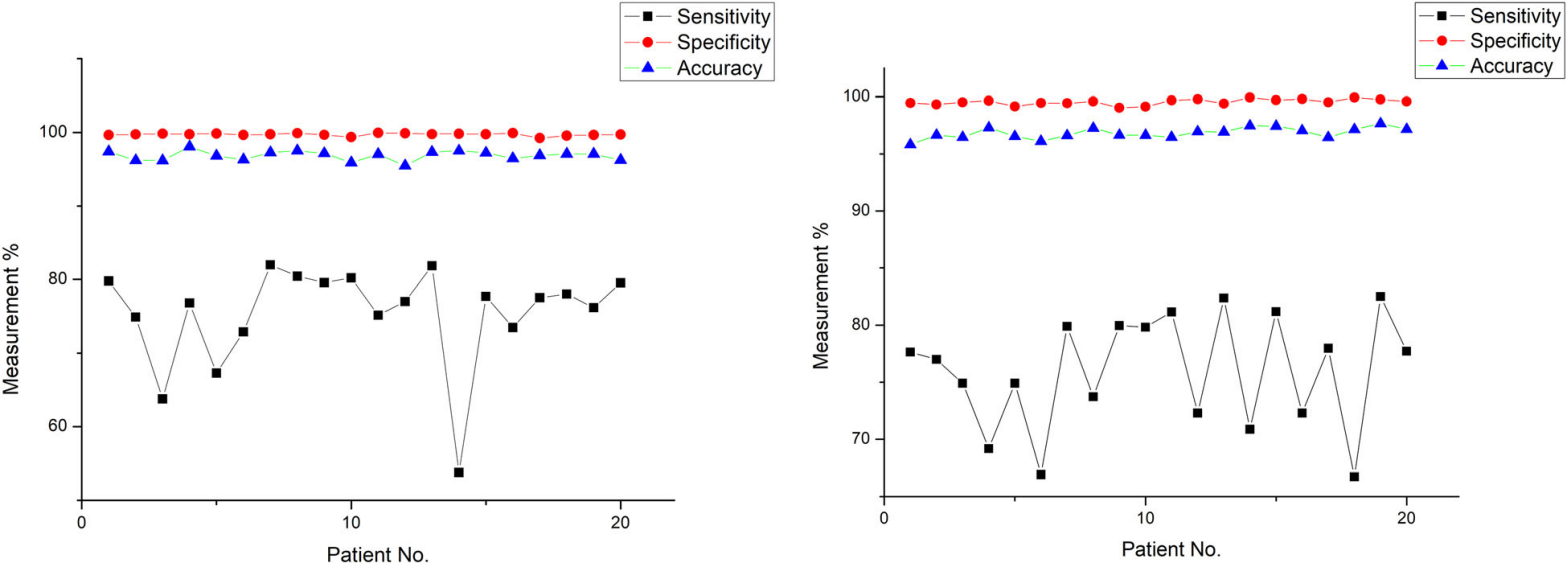
Results of sensitivity, specificity and accuracy for MRI images using the modified DRLSE for atrophy and normal subjects

Figures 7 and 8 show the measures used for assessing segmentation performance metrics such as sensitivity, specificity and accuracy for normal and atrophy subjects of CT and MR images respectively. It could be seen that the segmentation specificity (98%–99%) and accuracy (95%–98%) values were found to be higher for CT and MR images of normal and atrophy subjects. The image segmentation sensitivity values were found to be 90% for normal and atrophy subjects as shown in the Figs. 7 and 8. Further, it was observed that the lowest range of sensitivity for image segmentation was found to be low (46%).

**Fig. 9:**
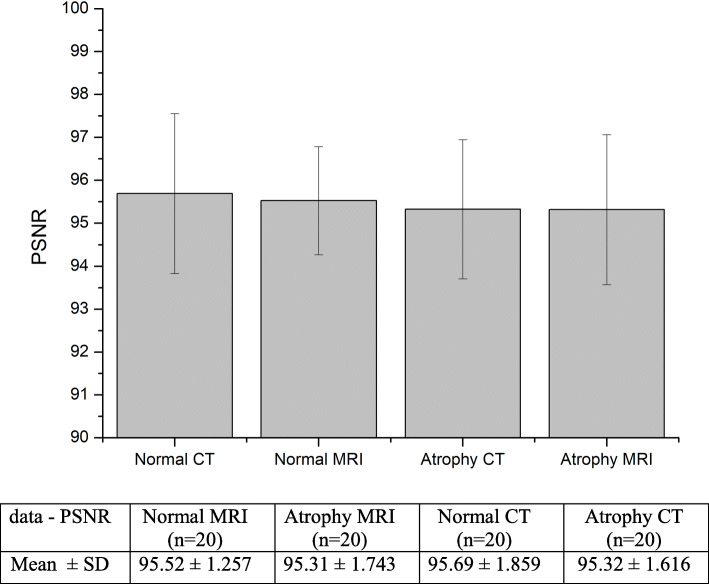
Results of PSNR calculated between ground truth and segmented images for CT and MR datasets

Figure 9 shows the results of PSNR estimated by comparing the segmented images with the ground truth images. The mean and SD for normal and atrophy groups of CT and MR images are shown in Fig. 9. Both MRI and CT images for normal and atrophy subjects resulted in PSNR mean value of 95%.

**Fig. 10:**
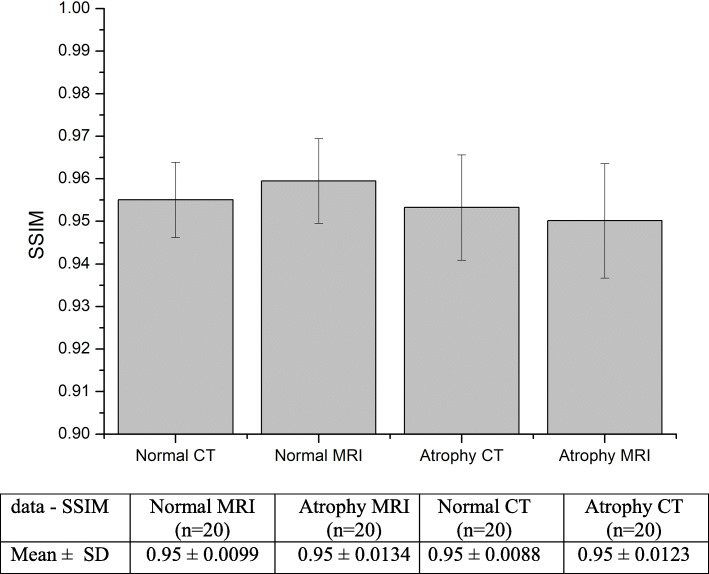
Results of SSIM calculated between ground truth and segmented images for CT and MR datasets

Figure 10 shows the results of the SSIM calculated between the segmented images and the ground truth images. The similarity index is measured between 0 and 1. If the SSIM is 0 there is no similarity and if it is 1 there is 100% similarity between the images. Mean and SD for normal and atrophy groups of CT and MR datasets are shown in Fig. 10. Both normal and atrophy groups of CT and MRI datasets yielded SSIM values of 0.95 with varying SDs.

Table 2 presents the comparison of results with recent papers, based on statistical analysis measures such as sensitivity, specificity and accuracy. In this study, few segmentation methods based on level set formulation and few based on other segmentation frameworks such as fuzzy based method, hybrid algorithm, and self-organizing map-based methods were included for comparison. The conventional DRLSE method was also implemented for the images and the results are presented in the Table 2.

**Table 2:** Results of the comparison of the proposed method with other segmentation methods from the literature

Methods	Sensitivity	Specificity	Accuracy
Proposed method (atrophy CT, *n* = 20; normal CT, *n* = 20; atrophy MRI, *n* = 20; normal MRI, *n* = 20)	65%–90%	98%–99%	95%–98%
DRLSE (implemented)	74%–79%	91%–93%	92%–94%
Region based level set (MRI, *n* = 124) [38]	84%–89%	96%–97%	–
DRLSE and deep structure (MRI, *n* = 45) [39]	–	–	84%–95%
Fuzzy and evidential reasoning (MRI, *n* = 17) [40]	55%–90%	–	60%–80%
Hybrid genetic and EM algorithm (MRI, *n* = 10) [41]	–	–	45%–85%
Bayesian morphometry (MRI, *n* = 95) [42]	60%–80%	30%–60%	–
EGS-SOM (MRI, *n* = 57) [43]	76%–80%	95%–96%	–
HFS-SOM (MRI, *n* = 57) [43]	66%–77%	80%–85%	–

It is evident from Table 2 that, the proposed DRLSE with RSF framework has yielded better results than other methods (overall based on all the three measures). Moreover, the proposed method is multimodal; performs well on CT and MR images. Accuracy (95%–98%) and specificity (98%–99%) are higher than all the other methods compared in this study. The sensitivity also falls in close range with other studies. The proposed modification was made in the external energy part that is derived from the image information (edge and regional intensity). Since the regularization term is not modified there will be no changes in the stability and re-initialization of the level set framework as DRLSE does not require re-initialization. All the methods shown in the table were applied on MR images.

## Discussion

Computer-aided diagnostic systems are required to perform quantitative characterization of medical images. This needs enhanced visualization which could be used for differential diagnosis. It is a common procedure to first extract the object of interest from these images to get a better visualization of the region of interest.

This paper presents a segmentation framework based on the modified DRLSE formulation for segmenting the ventricles from brain images of normal and atrophy subjects. Optimization was performed for the parameters that are involved in the segmentation algorithm for the given dataset. Figures 7 and 8 illustrate the segmentation results for CT and MRI datasets of normal and atrophy groups. Consistent results were obtained for both MRI and CT images containing normal and atrophy subjects. The specificity had the most consistent results across normal and atrophy subjects for CT and MRI dataset, with a SD of 0.1% to 0.3% while accuracy was the second most consistent with a SD in the scale of 0.4% to 0.9%. The sensitivity yielded the lowest values with a wide variation of 5% to 9%.

In addition to sensitivity, specificity, and accuracy measures, PSNR and SSIM were also measured to estimate the efficiency of the proposed method. PSNR values were in the range of –95% with SDs ranging from 1%–2%. SSIM also produced similar results in the range of 0.95 as shown in Figs. 9 and 10. It was observed that, the segmentation algorithm produced similar results for both groups. The proposed method was shown to perform better than other segmentation algorithms reported in the literature as shown in Table 2. The execution time for the proposed method takes up to 2 min for both CT and MR images.

## Conclusions

This study presents a level set based segmentation technique for segmenting ventricles from brain images for atrophy and normal subjects in CT and MRI datasets. The proposed method is based on DRLSE and RSF. The DRLSE method does not require re-initialization process, but it lacks the information on the regional intensity of the object being segmented. RSF derives its surface energy from the regional intensity information. The present study proposed a framework, combining the regional information from RSF with the conventional DRLSE method to improve its efficiency. The proposed method produced sensitivity in the range of 65%–90%, specificity in the range of 98%–99% and accuracy in the range of 95%–98%. PSNR and structural similarity index were also used to measure the segmentation accuracy and produced good results: 95% and 0.95 respectively. In addition to segmentation, an optimization procedure was carried out to fine tune parameters of DRLSE formulation for the input image dataset used. Presented method was found to be efficient and fast. The proposed method is adaptive for normal and atrophy subjects and is multi-modal. Future studies should involve analyzing shape differences of ventricles of normal and abnormal subjects for diagnosis and forming a generalized optimization technique.

## Data Availability

The datasets generated and/or analyzed during the current study are not publicly available due to ethical approval and policy but are available from the corresponding author on reasonable request.

## Abbreviations

CT: Computed tomography
MRI: Magnetic resonance imaging
PET: Positron emission tomography
SPECT: Single photon emission computed tomography
CSF: Cerebrospinal fluid
MCI: Mild cognitive impairment
DRLSE: Distance regularized level set evolution
LSF: Level set function
RSF: Region-scalable fitting
SD: Standard deviation
PSNR: Peak signal to noise ratio
SSIM: Structural similarity index measure
TN: True negative
TP: True positive
FN: False negative
FP: False positive

## References

[1] Gameraddin M, Alsayed A, Ali A, Al-Raddadi M (2015). Morphometric analysis of the brain ventricles in normal subjects using computerized tomography. Open J Radiol.

[2] Levine D, Trop I, Mehta TS, Barnes PD (2002). MR imaging appearance of fetal cerebral ventricular morphology. Radiology.

[3] Qiu W, Yuan J, Rajchl M, Kishimoto J, Chen YM, de Ribaupierre S (2015). 3D MR ventricle segmentation in pre-term infants with post-hemorrhagic ventricle dilatation (PHVD) using multi-phase geodesic level-sets. Neuroimage.

[4] Fabijańska A, Wegliński T, Zakrzewski K, Nowoslawska E (2014). Assessment of hydrocephalus in children based on digital image processing and analysis. Int J Appl Math Comput Sci.

[5] Ishii K, Kanda T, Harada A, Miyamoto N, Kawaguchi T, Shimada K (2008). Clinical impact of the callosal angle in the diagnosis of idiopathic normal pressure hydrocephalus. Eur Radiol.

[6] Damodara JA, Gangadhar BN (2012). Measuring complexity of lateral ventricle from MR images for schizophrenia. Abstracts of world congress on information and communication technologies.

[7] Anandh KR, Sujatha CM, Ramakrishnan S (2016). A method to differentiate mild cognitive impairment and Alzheimer in MR images using Eigen value descriptors. J Med Syst.

[8] Wang JN, Ekin A, De Haan G (2008). Shape analysis of brain ventricles for improved classification of Alzheimer's patients. Abstracts of the 15th IEEE international conference on image processing.

[9] Bader C, Cyrille C, Jadwiga Z, Joel D, Fichten A, Catherine GJ (2013). Estimation of the lateral ventricles volumes from a 2D image and its relationship with cerebrospinal fluid flow. Biomed Res Int.

[10] Carmichael OT, Thompson PM, Dutton RA, Lu A, Lee SE, Lee JY (2006). Mapping ventricular changes related to dementia and mild cognitive impairment in a large community-based cohort. Abstracts of the 3rd IEEE international symposium on biomedical imaging: nano to macro.

[11] Quigley SJ, Scanlon C, Kilmartin L, Emsell L, Langan C, Hallahan B (2015). Volume and shape analysis of subcortical brain structures and ventricles in euthymic bipolar I disorder. Psychiatry Res.

[12] Xia Y, Hu QM, Aziz A, Nowinski WL (2004). A knowledge-driven algorithm for a rapid and automatic extraction of the human cerebral ventricular system from MR neuroimages. Neuroimage.

[13] Gupta S, Singh YG (2009). Computer aided analysis of filters using level set segmentation for biomedical images.

[14] Mishra P, Agarwal S, Kiran U (2014). A hybrid technique of medical image segmentation using DRLSE algorithm. KrishiSanskriti Publ.

[15] Baghdadi M, Benamrane N, Sais L (2010). Segmentation of 3D brain structures using the Bayesian generalized fast marching method. Abstracts of international conference on brain informatics.

[16] Narr KL, Thompson PM, Sharma T, Moussai J, Blanton R, Anvar B (2001). Three-dimensional mapping of temporo-limbic regions and the lateral ventricles in schizophrenia: gender effects. Biol Psychiatry.

[17] Anandh KR, Sujatha CM, Ramakrishnan S (2014). Analysis of ventricles in Alzheimer MR images using coherence enhancing diffusion filter and level set method. Abstracts of IEEE 2014 international conference on informatics, electronics & vision.

[18] Angelini ED, Song T, Mensh BD, Laine AF (2007). Brain MRI segmentation with multiphase minimal partitioning: a comparative study. Int J Biomed Imaging.

[19] Aloui K, Naceur MS (2009). 3D brain tumor segmentation using level-sets method and meshes simplification from volumetric MR images. Int J Comput Inf Eng.

[20] D'Souza EDMC, Natekar PE (2007). Morphometric study of the ventricular system of brain by computerised tomography. J Anat Soc India.

[21] Li CM, Xu CY, Gui CF, Fox MD (2005). Level set evolution without re-initialization: a new variational formulation. Abstracts of 2005 IEEE computer society conference on computer vision and pattern recognition.

[22] Li CM, Xu CY, Gui CF, Fox MD (2010). Distance regularized level set evolution and its application to image segmentation. IEEE Trans Image Process.

[23] Malladi R, Sethian JA, Vemuri BC (1995). Shape modeling with front propagation: a level set approach. IEEE Trans Pattern Anal Mach Intell.

[24] Tsai R, Osher S (2003). Level set methods and their applications in image science. Commun Math Sci.

[25] Joshi N, Brady M (2010). Non-parametric mixture model based evolution of level sets and application to medical images. Int J Comput Vis.

[26] Vese LA, Chan TF (2002). A multiphase level set framework for image segmentation using the Mumford and Shah model. Int J Comput Vis.

[27] Osher S, Fedkiw RP (2001). Level set methods: an overview and some recent results. J Comput Phys.

[28] El Munim HEA, Farag AA (2007). Curve/surface representation and evolution using vector level sets with application to the shape-based segmentation problem. IEEE Trans Pattern Anal Mach Intell.

[29] Burger M, Osher SJ (2005). A survey on level set methods for inverse problems and optimal design. Eur J Appl Math.

[30] Paragios N, Paragios N, CHEN YM, Faugeras O (2006). Curve propagation, level set methods and grouping. Handbook of mathematical models in computer vision.

[31] Phillips CL (1999). The level-set method. MIT Undergrad J Math.

[32] Liu JQ, Liu WW (2011). Adaptive medical image segmentation algorithm combined with DRLSE model. Procedia Eng.

[33] Lu Z, Carneiro G, Bradley AP (2015). An improved joint optimization of multiple level set functions for the segmentation of overlapping cervical cells. IEEE Trans Image Process.

[34] Dongye CL, Zheng YG, Jiang DH (2014). A fast global minimization of region-scalable fitting model for medical image segmentation. J Softw.

[35] Yang YY, Li CM, Kao CY, Osher S (2010). Split Bregman method for minimization of region-scalable fitting energy for image segmentation. Abstracts of the 6th international conference on advances in visual computing.

[36] Li CM, Kao CY, Gore JC, Ding ZH (2008). Minimization of region-scalable fitting energy for image segmentation. IEEE Trans Image Process.

[37] Bai PR, Song DD, Bi LJ, Li L, Qi T (2015). A novel integration scheme based on mean shift and region-scalable fitting level set for medical image segmentation. Abstracts of the 2015 3rd international conference on machinery, materials and information technology applications.

[38] Yu CY, Zhang WS, Yu YY, Li Y (2013). A novel active contour model for image segmentation using distance regularization term. Comput Math Appl.

[39] Yang HZ, Zhao LH, Tang SY (2014). Brain tumor segmentation using geodesic region-based level set without re-initialization. Int J Signal Processing Image Processing Pattern Recognit.

[40] Ngo TA, Carneiro G (2014). Fully automated non-rigid segmentation with distance regularized level set evolution initialized and constrained by deep-structured inference. Abstracts of 2014 IEEE conference on computer vision and pattern recognition.

[41] Zhu HW, Basir O (2003). Automated brain tissue segmentation and MS lesion detection using fuzzy and evidential reasoning. Abstracts of the 10th IEEE international conference on electronics, circuits and systems.

[42] Herskovits EH, Peng H, Davatzikos C (2004). A Bayesian morphometry algorithm. IEEE Trans Med Imaging.

[43] Tian GJ, Xia Y, Zhang YN, Feng DG (2011). Hybrid genetic and variational expectation-maximization algorithm for Gaussian-mixture-model-based brain MR image segmentation. IEEE Trans Inf Technol Biomed.

